# Risk-taking behaviors and exposures among vocational school students in China: a cross-sectional survey

**DOI:** 10.5249/jivr.v12i3.1278

**Published:** 2020-08

**Authors:** Fenfen Li, Shumei Wang

**Affiliations:** ^*a*^ Ministry of Education-Department of Children and Adolescent Health, School of Public Health, Fudan University, Shanghai (200032), China.

**Keywords:** Vocational school, Injury, Risk behavior, Exposure

## Abstract

**Background:**

Injury has become the leading cause of death among adolescents. This study aimed to understand the prevalence of risk-taking behaviors and exposures and injuries among vocational school students and to provide guidance for further injury intervention.

**Methods:**

A cluster sampling method was used to conduct a questionnaire survey of all grade one and grade two students in a vocational school in Jiangsu Province in April 2018. A self-administered questionnaire was designed to investigate information on basic demographic information, risk-taking behaviors and exposures, and injuries.

**Results:**

A total of 1079 students were investigated, 490(45.41%) were boys; the mean age was 16.80±0.80 years; 560(51.90%) were grade one students. There were 57 injuries in the past 12 months. The overall injury incidence was 5.28%. The total proportions of risk behaviors ranged from 1.4% for playing on the street to 68.6% for not wearing reflective devices while walking at night. Behaviors of not using traffic safety protection devices were highly prevalent, including not using seat-belts in the back seat of cars (19.7%), not wearing helmet while taking/ riding an electric car (15.8%/13.0%), not wearing reflective devices while walking/riding at night (68.6% for pedestrians and 62.8% for two-wheelers riders). Girls generally had more negative mood exposures compared to boys. Boys generally had more traffic risk behaviors and bullying and violence experiences compared with girls. The average number of risk-taking behaviors and exposures was 8.73±8.06. The number of risk-taking behaviors and exposures was positively associated with injury, with OR of 1.07(1.04-1.10).

**Conclusions:**

Risk-taking behaviors and exposures were prevalent among vocational school students and increased the risk of injury. Traffic safety, bullying and violence, and sports safety were the aspects need more attention. The intervention of such risk behaviors should aim at the characteristics of the population and their special behavior problems.

## Introduction

With the society developing and medical advancing, traditional infectious diseases and other common diseases have been effectively controlled. Injury has become the leading cause of death among adolescents in the world.^1,2^ According to the WHO report in 2017, among the top five deaths of young people aged 10-19 in the Western Pacific Region, road traffic injuries ranked first, drowning ranked second, and self-harm ranked fourth.^1^ Road traffic injuries and self-harm were the 1st and the 5th reasons for DALYs.^3^ Report on the Status of Injury of Young Children in China in 2017 stated that almost 55 thousand of children and adolescents in China died of injuries each year, and 132 thousand received emergency treatment due to injury.^2^

Most unintentional injuries are preventable. Among adolescents, risk-taking behaviors and other exposures may be a significant contributor to the injury rate. Those behaviors and exposures include incorrect road using behaviors, being physically attacked, being in a physical fight, being bullied, using tobacco, drinking alcohol, sexual intercourse, anxiety, insomnia, and loneliness.^4^ Many risk behaviors and exposures linked to injuries can be effectively addressed through effective interventions. The identification of the specific behaviors and exposures in various populations is an essential step for the development and implementation of effective injury prevention programs and interventions.^5^

Vocational school students were a special population among teenagers in China. The majority of them were those underachievement students in junior high school. For those students, they may be more likely to get involved in bullying, violence, and other risk-taking behaviors and exposures. The purpose of this study was to understand the prevalence of risk-taking behaviors and exposures and injuries among vocational school students, analyze the association between risk-taking behavior and exposures and injuries, and to provide guidance for further injury intervention.

## Methods 

**Design and Participants**

A cluster sampling method was used to select all the grade one and grade two students in a vocational school in a community of Jiangsu Province in China as the participants in April 2018. The grade three students were out of school for their professional practice and were not available for the investigation. 

**Questionnaire development and Assessment**

Following the review of literature and consultation with researchers and health professionals, a short self-administered questionnaire was designed to investigate the basic demographic information, injuries and risk-taking behaviors and exposures, such as traffic risk behaviors, violence/bullying exposures, unsafe exercising behaviors, depression. A total of 49 questions on risk-taking behaviors and exposures were investigated. Each question had three levels of “often”, “rarely”, and “never”. The questionnaire was further evaluated and modified by experts in the field of epidemiology, injury prevention mapping, health education, and fall prevention professionals for its content validity and clarity. The reliability score of the questions of risk-taking behaviors and exposures by Cronbach's alpha test was 0.93. To facilitate the analysis, for each question the answer of “often” and “rarely” was set as “yes” and “never” was set as “no”. The “yes” answers of all the questions were counted up as a new variable of the number of risk-taking behaviors and exposures, with a higher number representing worse behavior. 

**Data collection**

The data was collected by an electronic online questionnaire. The students were all informed of the purpose and meaning of the survey. Quality control was conducted during the questionnaire design, data collection, and post-investigation. During the questionnaire design, quality control methods included setting a questionnaire entry password and setting reverse questions and jumping questions in the questionnaire. In the data collection period, the investigation was conducted by trained class teachers and the investigators, with a class as a unit to fill out the questionnaire in the school computer classroom. Any related questions in the process of investigation would be answered by the trained class teachers and the investigators. When the data was collected, the data was checked and the illogical results and the unqualified questionnaires were eliminated to ensure the data quality. 

**Statistical analysis**

All statistical analyses were performed with the SPSS V22.0 software package. Continuous variables were expressed as mean±SD and categorical data as proportion. Independent-samples t-tests were performed to compare HBM dimension scores according to demographic variables. Binary logistic regression analysis was conducted to analyze the association between risk-taking behaviors and exposures and injury. Odds ratios (ORs) and 95% confidence intervals (CIs) were calculated. An alpha level of less than 5% was considered to be statistically significant.

## Results

A total of 1229 student questionnaires were distributed, and 1079 valid questionnaires were collected. Among the 1079 students, 490(45.41%) were boys; the mean age was 16.80±0.80 years; 560(51.90%) were grade one students (Table 1). There were 57 injuries in the past 12 months. Thirteen of them were traffic injuries, accounting for 22.81% of all the injuries. The overall injury incidence was 5.28% and traffic injury incidence was 1.21% (Table 2).

**Table 1:** Characteristics of the 1 079 students and injuries in the past 12 months.

Variable	Total	Injury	Traffic injury
Gender, N/%			
Boy	490(45.41%)	28(49.12%)	8(61.54%)
Girl	589(54.59%)	29(50.88%)	5(38.46%)
Age (years), mean±SD	16.80±0.80	16.30±0.82	16.46±0.88
Grade, N/%			
Grade one	560(51.90%)	18(31.58%)	5(38.46%)
Grade two	519(48.10%)	39(68.42%)	8(61.54%)
**Total**	**1079(100%)**	**57(5.28%)**	**13(1.21%)**

**Table 2:** Risk-taking behaviors and exposures of the 1 079 students.

Content	Boy	Girl	Total
1. Crossing a road barrier	4.5	0.7	2.4
2. Running a red light	4.5	0.8	2.5
3. No pedestrian crossing or footbridge	5.7	1.0	3.2
4. Playing with mobile phones while crossing the street	8.0	5.6	6.7
5. Playing on the street	3.1	0.0	1.4
6. Playing with mobile phones or listen to music while walking.	17.3	15.3	16.2
7. Take people by bike, both hands away from the handle, etc.	5.5	0.2	2.6
8. Chase, zigzag, or drag racing by bike	3.7	0.2	1.8
9. Chase, zigzag, or drag racing by electric vehicles	4.5	0.3	2.2
10. Not using seat-belts in the back seat of cars	20.0	19.5	19.7
11. Not using seat-belts in car copilot	6.3	2.0	4.0
12. Not wearing a helmet while taking an electric car	18.0	14.1	15.8
13. Not wearing a helmet while riding an electric car	16.7	9.8	13.0
14. Using a cell phone or other electronic device while riding an electric bicycle	6.9	1.4	3.9
15. Not wearing reflective devices while walking at night	70.2	67.2	68.6
16. Not wearing reflective devices while riding two-wheelers at night	64.5	61.5	62.8
17. being in a car driven by a drinker	3.7	0.3	1.9
18. Parents using mobile phones while driving a private car	29.6	29.4	29.5
19. Taxi or bus drivers using mobile phones while driving	23.9	23.9	23.9
20. Do not want to go to school	38.0	43.3	40.9
21. Been maliciously teased	26.5	21.2	23.6
22. Been claimed for property	11.0	3.4	6.9
23. Been excluded on purpose by classmates	15.1	13.4	14.2
24. Someone did erotic teasing or pornography for you	28.2	8.7	17.5
25. Been harassed in public places	11.0	3.6	7.0
26. Been teased for physical defects or appearance	15.1	9.5	12.0
27. Been threatened or intimidated	10.0	3.4	6.4
28. Been beaten, kicked, pushed, squeezed or locked up in the house	10.2	2.2	5.8
29.Someone used electronic media to tease, abuse, threaten, spread rumors about you	9.4	3.7	6.3
30. Witnessed home violence	16.1	7.3	11.3
31. Been spanked by parents	16.1	13.9	14.9
32. Not holding the handrail and looking at the cellphone while taking the elevator	25.5	31.6	28.8
33. No preparing activities before sports	38.4	43.8	41.3
34. No protective measures such as kneecaps during exercise	36.9	40.7	39.0
35. Playing with domestic pet	28.6	30.1	29.4
36. Playing with stray cats	18.8	20.0	19.5
37. Eating food from street vendors	45.1	52.6	49.2
38. Got battery cars charged in the corridors	14.5	6.1	9.9
39. Jumbling the wires in the house	13.7	4.6	8.7
40. Swimming in non-swim areas	10.0	2.0	5.7
41. Smoking	13.5	3.9	8.2
42. Drinking	31.4	24.8	27.8
43. Take addictive drugs	7.6	1.0	4.0
44. Surfing the Internet at Internet Cafes	32.0	7.3	18.5
45. Fighting with someone	16.5	2.7	9.0
46. Been unpleasant because of stress and achievements of study	31.2	41.4	36.8
47. Suffering from insomnia	33.5	40.2	37.2
48. Had depression symptoms	25.5	25.0	25.2
49. Have encountered inextricable problems and felt desperate	25.7	26.5	26.1

Note: Data were expressed as proportions (%).

Table 2shows the proportions of the 49 risk-taking behaviors and exposures in the past 12 months. The total proportions of risk behaviors ranged from 1.4% playing on the street to 68.6% for not wearing reflective devices while walking at night. Behaviors of not using traffic safety protection devices were highly prevalent, including not using seat-belts in the back seat of cars (19.7%), not wearing helmet while taking/riding an electric car (15.8%/ 13.0%), not wearing reflective devices while walking/riding at night (68.6% for pedestrians and 62.8% for two-wheelers). Girls generally had more negative mood exposures compared to boys. Boys generally had more traffic risk behaviors and bullying and violence exposures compared with girls.

The average number of risk-taking behaviors and exposures was 8.73±8.06. The number was 9.72±10.03 for boys, 7.91±5.83 for girls, 8.73±7.91 for grade one students and 8.74±8.22 for grade two students. Compared to girls, boys presented more risk behaviors (p<0.001). There was no difference between grade one and grade two students (p>0.05). Table 3 shows the binary logistic regression analysis of the number of risk-taking behaviors and exposures and injury among vocational school students. The number of risk-taking behaviors and exposures was positively associated with injury, with OR of 1.07(1.04-1.10) for both none-adjusted model and adjusted model.

**Table 3:** Binary logistic regression analysis of the number of risk-taking behaviors and exposures and injury.

Models	β	Sβ	Wald	p-value	OR（95%CI）
Model 1: None-adjusted model	0.064	0.012	27.700	<0.001	1.07(1.04-1.10)
Model 2: Adjusted model	0.066	0.013	27.034	<0.001	1.07(1.04-1.10)

Note: Variables of gender and grade were adjusted in adjusted model.

## Discussion

This study investigated the risk-taking behaviors and exposures and injuries among vocational school students. Risk-taking behaviors and exposures increased the risk of injury, with OR of 1.07. The overall injury incidence was 5.28% and was higher than that in other studies among middle and high school students.^6,7^ The total proportions of risk behaviors ranged from 1.4% to 68.6%. The average number of risk-taking behaviors and exposures was 8.73. The incidences of risk-taking behaviors and exposures were high. The study results showed that vocational school students had a high prevalence of traffic risky behaviors, especially of not using traffic safety protection devices, bullying/violence, sports risk behaviors, and unsafe animal contact. Girls reported more mental exposures, and boys reported more involvement in physical behaviors, which was the same as the results in other studies.^4^

The use of helmets, seat-belt, reflective equipment, and other traffic safety protection devices reduced the severity of injuries in traffic accidents and was of great significance for safety. In this study, traffic safety protection devices were at low rates of using. Thus, the students were likely to become potential victims of traffic injuries. Considering that the vocational school students had a high rate of electric bicycles with high speed, parents need to assist their children to purchase and use the safety protection devices, such as helmets and reflective strips. Safety education targeted on different stakeholders of the students, families, schools, and communities should be strengthened. In addition, the traffic management department should strengthen the legislation and implementation of laws on traffic behaviors, strengthen traffic management, and create a good community traffic environment.^8^

Bullying and violence increased the risk of injury.^4^ It affects the physical and mental health of the involved students. Bullying and violence exposures were ranged from 6.3% to 40.9% in this study. Generally, for those underachievement students in junior high school, they cannot enter a senior high school because of the poor graduation examination results. The majority of those students would choose a vocational school to learn certain techniques. They were the “poor students” when they were in junior high school or even primary school and were more likely to get involved in bullying. Besides, the education goal of vocational schools was to train special skills for getting a job. The teaching mode was similar to university education. Students were in the transitional stage from campus to society. However, the average age of the students was smaller and they were generally underage. They were prone to have some behavioral and psychological problems in such transition, affecting the physical and mental development, socialization and social adaptation in future. ^9^ It is recommended that schools strengthen the construction of an excellent campus atmosphere and strengthen the ability of teachers in detecting and responding to school bullying. ^10^ Schools should also establish bullying prevention schemes and disposal regulations.

Sports safety protection measures reduced the incidence of injury during the student's exercise and reduce the severity of the injury when the injury occurs, such as adequate preparation and wearing protective devices before exercise;^11^ however, they were not in place. About 40% of the students had no preparing activities nor kneecaps. Of the activity in which injury occurred, 14.4% were extracurricular sports activities, and 13.6% were in sports lessons (data from the injury survey results of the same population). The school should standardize students' sports lessons and sports safety manuals. Physical education teachers should strictly carry out sports activities in accordance with the course safety requirements, strengthen regular sports safety lectures or theoretical physical education classes, and learn from foreign effective measures, such as signing Safety Sports Protocol.^12^

Individual behaviors and exposures were influenced by individuals themselves, families, schools, and the social environment. The intervention strategies should make full use of the resources to solve the obstacles in behavior intervention and create a safe atmosphere in the society, to make the related behaviors becoming social norms like no-drinking and no-smoking.^13^

## Conclusion

Risk-taking behaviors and exposures were prevalent among vocational school students and increased the risk of injury. Traffic safety, bullying and violence exposures, and sports safety were the aspects need more attention. The intervention of such risk behaviors should take into consideration of the population characteristics and their special behavior problems.

**Acknowledgment**

**Author Contributions:** Concept and design: All authors. Acquisition, analysis, or interpretation of data: All authors. Drafting of the manuscript: Li. Critical revision of the manuscript for important intellectual content: Wang. Statistical analysis: Li. Administrative, technical, or material support: Wang. We thank all the research staff and all the participants for their dedication to this study.

**Appendix:** Chinese version of the questionnaire.

一、基本情况			
1. 出生日期：_______年_____月_____日			
2. 性别：⑴男 ⑵女			
3 年级：_______			
二、伤害发生情况			
1. 在过去一年中，你发生过伤害事故吗？			
⑴有 ⑵没有			
2．如果有，一共发生过___________次？			
3. 伤害性质是什么？			
⑴交通事故 ⑵跌落/跌倒 ⑶物体或人撞击/打击 ⑷刺或切割 ⑸枪击 ⑹火灾、火焰、热烫物质 ⑺窒息或悬吊 ⑻淹溺 ⑼电击或辐射 ⑽中毒 ⑾医疗或手术并发症 ⑿其他___________			
三、伤害相关危险行为和暴露			
			
危险行为和暴露	经常	偶尔	从不
1. 翻越道路隔离杆或隔离栏过马路	(1)	(2)	(3)
2. 过马路闯红灯	(1)	(2)	(3)
3. 过马路不走人行横道线或行人天桥	(1)	(2)	(3)
4. 过马路时玩手机或其他电子设备	(1)	(2)	(3)
5. 在马路上打闹	(1)	(2)	(3)
6. 一边走路一边玩手机或听音乐	(1)	(2)	(3)
7. 骑自行车带人、双手离把、攀附机动车	(1)	(2)	(3)
8. 骑自行车相互追逐或曲折竞驶、飙车	(1)	(2)	(3)
9. 骑电动车相互追逐或曲折竞驶、飙车	(1)	(2)	(3)
10. 乘坐轿车后排不系安全带	(1)	(2)	(3)
11. 乘坐轿车副驾驶位不系安全带	(1)	(2)	(3)
12. 乘电动车不戴头盔	(1)	(2)	(3)
13. 骑电动车不戴头盔	(1)	(2)	(3)
14. 骑电动车时使用手机或其他电子设备	(1)	(2)	(3)
15. 晚上步行出行不穿戴或使用反光条或有反光功能的设备	(1)	(2)	(3)
16. 晚上骑两轮车出行不穿戴或使用反光条或有反光功能的设备	(1)	(2)	(3)
17. 乘坐过喝酒者开的轿车	(1)	(2)	(3)
18. 乘坐私家车，父母或家人开车过程中使用手机通话或发信息	(1)	(2)	(3)
19. 乘坐出租车或公交车，司机开车过程中使用手机通话或发信息	(1)	(2)	(3)
20. 感觉不喜欢上学	(1)	(2)	(3)
21. 曾经被恶意取笑	(1)	(2)	(3)
22. 曾经被索要财物	(1)	(2)	(3)
23. 曾经被同学故意排斥	(1)	(2)	(3)
24. 曾经有人对你开色情玩笑或做色情动作	(1)	(2)	(3)
25. 在公共交通工具或场所遭遇性骚扰	(1)	(2)	(3)
26. 曾经因身体缺陷或长相而被取笑	(1)	(2)	(3)
27. 曾被威胁、恐吓	(1)	(2)	(3)
28. 曾被打、踢、推、挤或关在屋里	(1)	(2)	(3)
29. 有人通过电子传媒方式恶意取笑、谩骂、威胁、恐吓或者散布有关你的谣言、影像或视频等	(1)	(2)	(3)
30. 曾经目睹过家庭暴力	(1)	(2)	(3)
31. 被父母体罚、打骂或是其他方式惩罚过	(1)	(2)	(3)
32. 乘电梯时看手机不扶电梯扶手	(1)	(2)	(3)
33. 体育运动前未做准备活动	(1)	(2)	(3)
34. 运动时没有采取防护措施	(1)	(2)	(3)
35. 三与家养犬或猫咪等宠物玩耍	(1)	(2)	(3)
36. 与流浪猫等玩耍	(1)	(2)	(3)
37. 吃过路边摊食物	(1)	(2)	(3)
38. 在楼道内给电瓶车拖线充电	(1)	(2)	(3)
39. 在住房内乱拉乱接电线	(1)	(2)	(3)
40. 曾经到非泳区游泳	(1)	(2)	(3)
41. 有过吸烟行为	(1)	(2)	(3)
42. 有过饮酒行为	(1)	(2)	(3)
43. 有过成瘾药品使用行为	(1)	(2)	(3)
44. 曾在网吧上网	(1)	(2)	(3)
45. 曾打过架	(1)	(2)	(3)
46. 因学习压力或成绩问题而心情不愉快	(1)	(2)	(3)
47. 有失眠	(1)	(2)	(3)
48. 抑郁症状	(1)	(2)	(3)
49. 曾遇到解决不了的问题，无人可以倾诉，绝望无助	(1)	(2)	(3)
